# Electoral and religious correlates of COVID-19 vaccination rates in Dutch municipalities

**DOI:** 10.1093/eurpub/ckac112

**Published:** 2022-08-23

**Authors:** Alexandre Afonso, Fabio Votta

**Affiliations:** Institute of Public Administration, Faculty of Governance and Global Affairs, Leiden University, The Hague, The Netherlands; Amsterdam School of Communication Research, University of Amsterdam, Amsterdam, The Netherlands

## Abstract

Vaccination campaigns amid the coronavirus disease 2019 (COVID-19) pandemic have been extensively politicized in a number of countries. Controlling for a number of demographic, social and economic factors, we find a negative statistical relationship between the aggregate vote share of the populist-right wing Forum for Democracy and the vaccination rate against COVID-19 across Dutch municipalities. We also find a negative relationship between the proportion of individuals with reformed Protestant and Muslim religious beliefs. These relationships can possibly be related to religious worldviews or mistrust towards authority. These results show that the politicization of health behaviours can have detrimental effects on public health campaigns.

## Introduction

Countries around the world have rolled out extensive vaccination programmes to combat the coronavirus disease 2019 (COVID-19) pandemic. Yet, vaccination rates against COVID-19 still vary significantly across and within countries.^1^ Some of these differences can be explained by economic resources and differential access to vaccines, but others relate to individual attitudes and beliefs. Because vaccination programs in most countries are voluntary, it is important to understand which of these beliefs (e.g. political or religious) correlate positively or negatively with vaccination rates. This short report provides an analysis of the correlates of vaccination rates across Dutch municipalities made public by the Dutch public health authority (RIVM), paying particular attention to political and religious factors.

The Netherlands is an interesting case because of the historically high level of vaccine hesitancy among certain religious groups, namely Orthodox Protestants.^2^ Besides, COVID-19 vaccination in the Netherlands has been vividly politicized. Vaccination remained voluntary—no universal vaccination mandates were passed—but government parties have encouraged it while one party in particular [the radical right *Forum for Democracy* (FvD)] opposed what it perceived as a campaign of forced vaccination, notably through a vaccine or negative test requirements to access certain venues, such as restaurants. If the role of partisanship in vaccine hesitancy rates has been documented in the politically polarized context of the USA,^3^^,^^4^ we lack empirical evidence on whether partisanship shapes vaccination rates in European countries.

## Methods

We use data on vaccination rates per municipality on 29 December 2021 made available by the Dutch National Institute for Public Health. We use the share of people above 18 having received a full course of vaccination (two doses at the time).^5^ For religious affiliation, we use data from the Dutch Central Bureau for Statistics from a national enquiry on religion and church attendance per municipality conducted between 2010 and 2014.^6^ Unfortunately, more recent data at the local level were not available. Data on aggregate vote shares of Dutch political parties across Dutch municipalities in the 15–17 March 2021 parliamentary election were collected from the Dutch election authority.^7^ Socio-economic and demographic data come from the ‘core numbers cities and neighbourhoods’ 2019 collected by the Central Bureau for Statistics.^8^ More recent data only contained a smaller number of variables, and we used the 2019 edition to include a broader range of covariates.

We use ordinary least square (OLS) regressions regressing the vaccination rate per municipality (*n* = 327) on religious, political and socio-demographic indicators. There are 344 Dutch municipalities, and 17 of them were left out due to missing data. We estimate four models: Model 1 only contains socio-demographic controls; Model 2 contains religious variables and the controls, Model 3 contains the political variables and controls, and Model 4 contains all the variables. For a full overview of descriptives, bivariate correlations and summary statistics, see Supplementary files.

## Results

We report the OLS results in table 1. Model 1, which includes only socio-demographic controls, already explains 36.6% of the variance of the vaccination rate at the municipality level (adj. *R*^2^ = 0.366). Adding religious indicators (Model 2) adds substantially to the explanatory power, explaining 50.7% of the variance (adj. *R*^2^ = 0.507). Adding political indicators (Model 3) leads to an even greater share of explained variance: 84.5% (adj. *R*^2^ = 0.845). The final model (Model 4), including all variables, explains 86.7% of the variance (adj. *R*^2^ = 0.867). This underlines the importance of political variables to account for the variance in local vaccination rates. We now focus on interpreting the results. Reported results are from Model 4, unless otherwise specified.

**Table 1:** OLS models

	Dependent variable: % fully vaccinated (18+)			
	(Model 1)	(Model 2)	(Model 3)	(Model 4)
Inhabitants (1000 s)	−0.015^***^	−0.012^**^	−0.011^***^	−0.006^**^
	(0.004)	(0.004)	(0.002)	(0.002)
% Men	2.226^***^	**0.705^+^**	−0.063	−0.498^*^
	(0.385)	(0.411)	(0.210)	(0.222)
% Elderly (65+)	0.624^***^	0.185^*^	0.189^***^	0.025
	(0.089)	(0.091)	(0.052)	(0.053)
% Highly educated	0.434^***^	0.299^***^	−0.001	−0.049
	(0.070)	(0.065)	(0.052)	(0.050)
Avg. household income	0.254^*^	0.130	−0.223^*^	−0.179^*^
	(0.125)	(0.112)	(0.086)	(0.083)
% Protestant		0.060		0.035
		(0.041)		(0.028)
% Reformed		−0.389^***^		−0.074^*^
		(0.051)		(0.037)
% Muslim		−0.469^***^		−0.440^***^
		(0.113)		(0.060)
% Catholic		(0.015)		0.002
		(0.015)		(0.012)
% Government parties (VVD, CDA, D66, CU)			0.347^***^	0.282^***^
			(0.037)	(0.046)
% PVV (populist right)			0.269^***^	0.243^**^
			(0.065)	(0.082)
% JA21 (populist right)			−0.091	−0.197
			(0.213)	(0.208)
% FvD (populist right)			−1.636^***^	−1.692^***^
			(0.157)	(0.163)
% SGP (orthodox protestant)			−0.451^***^	−0.498^***^
			(0.032)	(0.047)
Observations	327	327	327	327
*R* ^2^	0.376	0.521	0.849	0.873
Adjusted *R*^2^	0.366	0.507	0.845	0.867

*Notes*: +*P* < 0.1;

* *P* < 0.05;

** *P* < 0.01;

*** *P* < 0.001; statistically significant results in bold.

### Socio-demographic variables

Larger municipalities have on average a smaller share of vaccinated people, a finding that is consistent across all four models (*b* = −0.006; SE = 0.002; *P* < 0.01). The share of men and average household income is positively associated with vaccination rates in Model 1; however, the coefficients reverse when controlled for both religious and political indicators (Model 4). The share of elderly as well as highly educated people in a municipality is associated with higher vaccination rates in Model 1; however, the effect vanishes when controlled for religious and political indicators (Model 4).

### Religious variables

The share of protestants in a municipality is not associated with vaccination rates (*b* = −0.035; SE = 0.028; *P* > 0.05). The share of members of reformed protestant churches, a subset of protestant churches with more orthodox views, however, is associated with lower vaccination rates in Model 3. The effect is greatly reduced but still significant when controlling for political variables in Model 4. For every percent of Muslims in a municipality, the vaccination rate is 0.440 percentage points lower (*b* = −0.440; SE = 0.060; *P* < 0.001). The share of Catholics is positively associated with vaccination rates; however, the effect vanishes when controlled for political variables.

### Electoral variables

The current coalition government in the Netherlands consists of the VVD (centre-right), D66 (centre-left), CDA and *ChristenUnie* (Christian democrats), and we grouped the vote share of these parties together. The share of support for the government parties is positively associated with local vaccination rates (*b* = 0.282; SE = 0.046; *P* < 0.001). Next, we look at the relationship between the vote share of different radical-right parties and the vaccination rate as these parties adopt a critical stance towards government policy, possibly spurring vaccine hesitancy. Support for the populist-right PVV is also associated with higher levels of vaccinations (*b* = 0.243; SE = 0.082; *P* < 0.01). Support for the populist-right JA21 is not associated with vaccination rates, yet the populist-right FvD, which has taken clear positions against vaccines, on the other hand, is strongly associated with lower vaccination rates: for each percentage point vote share for the FvD, the vaccination rate is 1.69 percentage points lower (*b* = −1.692; SE = 0.163; *P* < 0.001). Support for the orthodox-protestant SGP is also associated with lower vaccination rates (*b* = −0.498; SE = 0.047; *P* < 0.001). The reduction in the coefficient of reformed protestants in the full model may be due to the inclusion of the vote share of the SGP, which sources most of its vote share in this religious community.

### Robustness tests

We estimate specification curves^9^ to test for the robustness of our findings and report the results in the Supplementary materials. The majority of our findings are replicated using different specifications (i.e. including different variables in the OLS). We find that income as well as PVV vote shares do not have a robust effect on vaccination rates in either direction. For more details, consider the Supplementary materials.

## Discussion

The results presented here show a clear and robust negative correlation between the vote share of a party opposing vaccinations and effective vaccination rates against COVID-19. The analysis using aggregate data should be considered with the usual caveats regarding possible ecological fallacies; observational data are also subject to the usual caveats about causality. It is unclear whether the messaging of parties creates vaccine hesitancy or whether people who are distrustful of vaccines are also more likely to vote for populist-right parties like the FvD. The implications of our findings are significant for public health. While existing research on the Dutch case had documented the importance of religious beliefs in vaccine take-up, a factor that we still find to be significant, we have found that some political orientations now also correlate with vaccination rates. Our findings highlight the importance of looking at politics and partisanship in explaining health and health behaviours.^10^ The politicization of health decisions such as vaccination choices can lead to outcomes that are detrimental to public health campaigns.

## Supplementary data

Supplementary data are available at *EURPUB* online.

## Funding

This research benefitted from funding from the Dutch Research Council NWO, Grant nr. 016.Vidi.185.159.

*Conflicts of interest*: None declared.

Key points

- We report a robust negative relationship between the vote share of a political party opposing vaccinations and coronavirus disease vaccination rates across Dutch municipalities.
- We also find a negative relationship between the proportion of adherents of certain religious denominations (reformed protestant and Muslim) and vaccination rates.
- In the context of politicization, partisanship should be taken seriously to understand health behaviours and public health outcomes.

## Supplementary Material

ckac112_Supplementary_Data
