# Supplementary material for: Electoral and religious correlates of COVID-19 vaccination rates in Dutch municipalities
Source: Eur J Public Health. 2022 Aug 23;32(6):985–7. doi: 10.1093/eurpub/ckac112 (PMC9452168; doi:10.1093/eurpub/ckac112)

Supplementary Materials

# 1 Bivariate Correlations and Scatter Plots

## 1.1 Party Vote Share

### 1.1.1 Party Vote Share X COVID Vaccination Rates


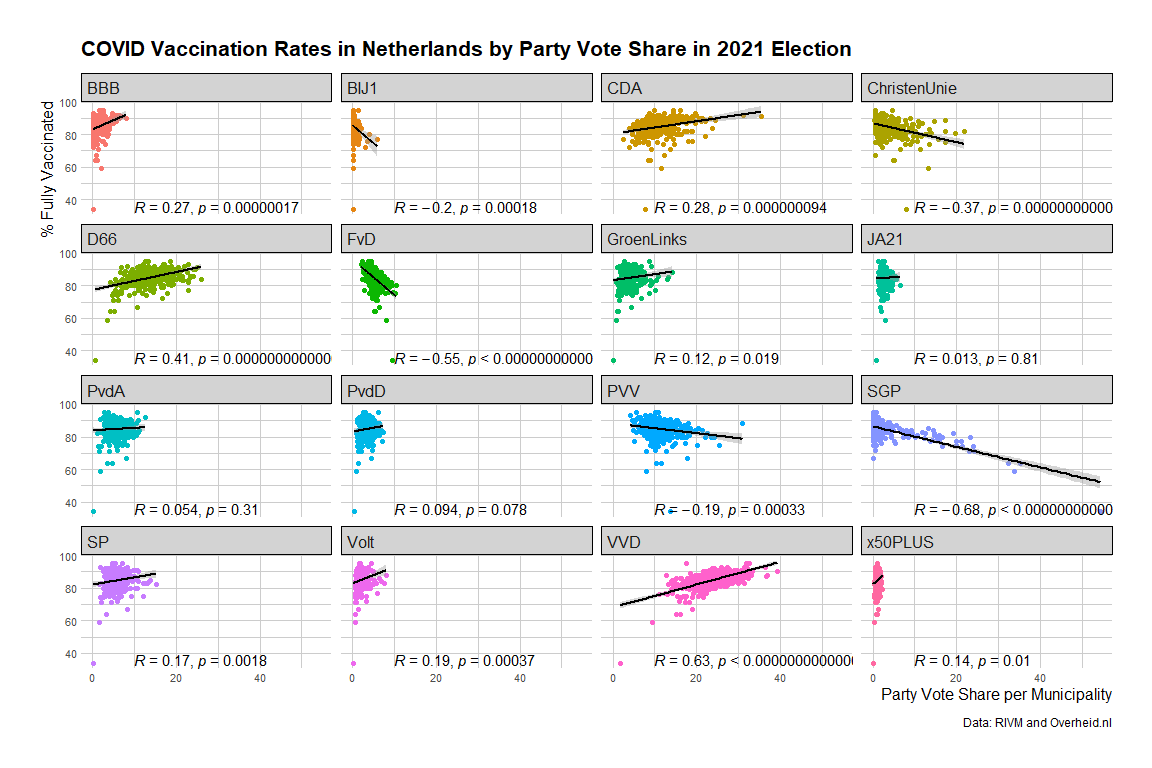


### 1.1.2 Party Vote Share X COVID Vaccination Rates (without outliers)

Shaping off 5% of top -and below distribution for each party


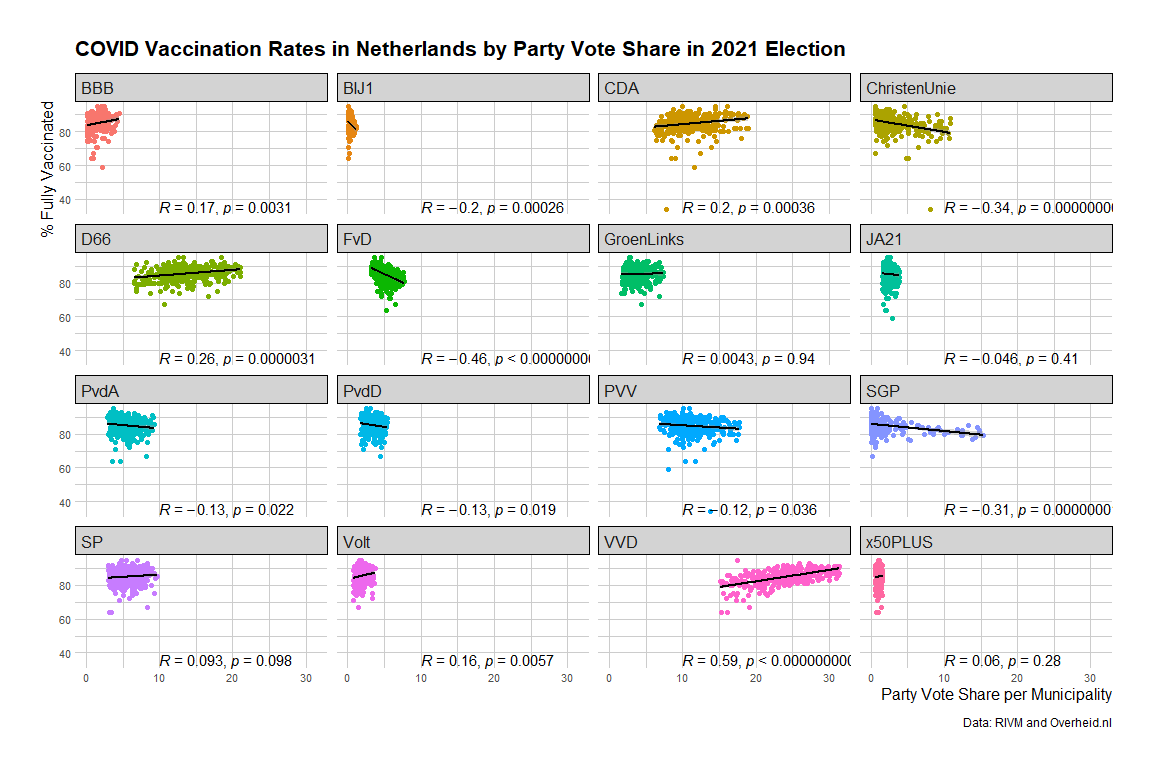


## 1.2 All Other Variables (Demographics, Religion etc.)

This includes population, share of men, and old people etc.

### 1.2.1 Demographics X COVID Vaccination Rates


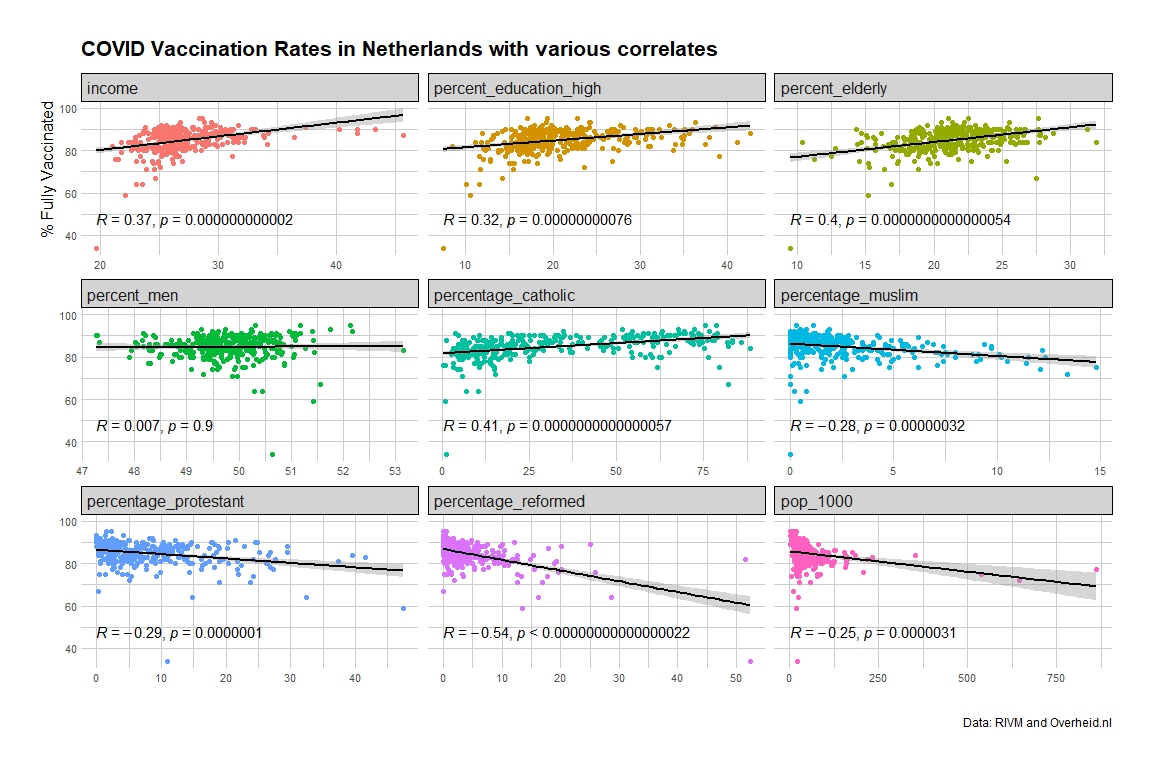


### 1.2.2 Demographics X COVID Vaccination Rates (without outliers)

Shaping off 5% of top -and below distribution for each variable


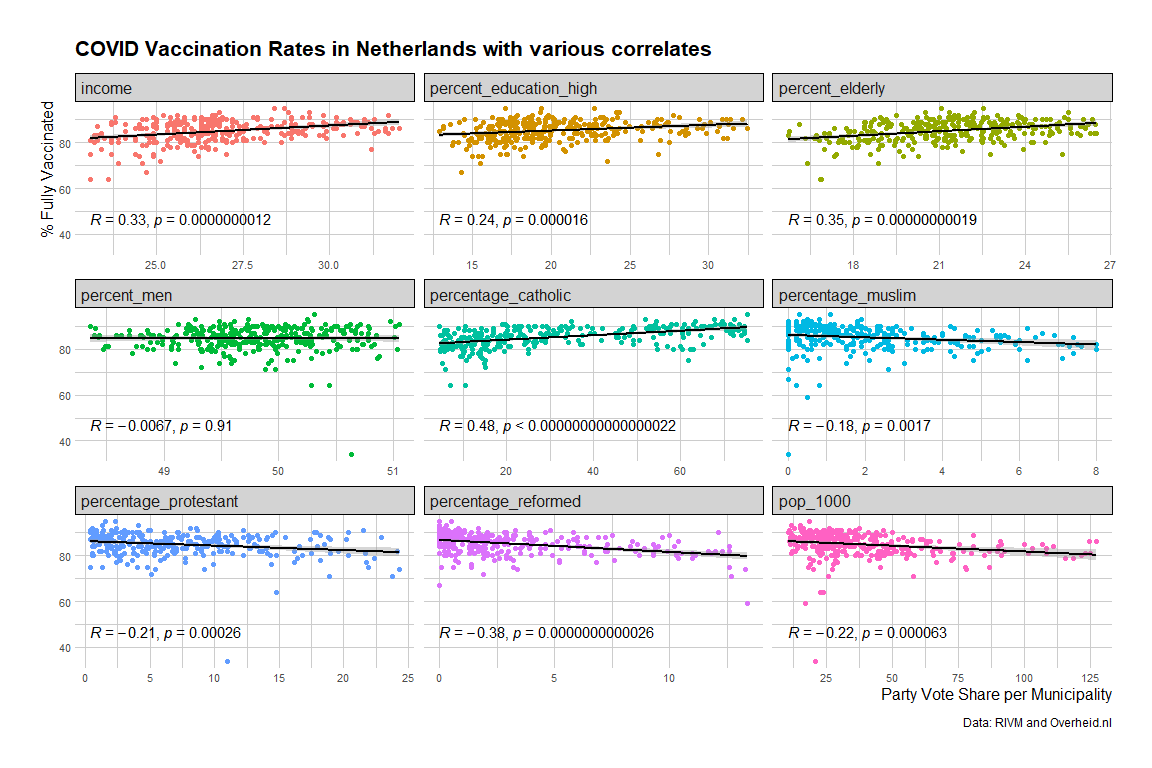


# 2 Analysis

## 2.1 Model 1


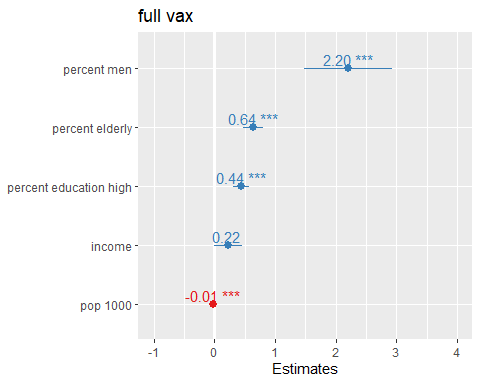


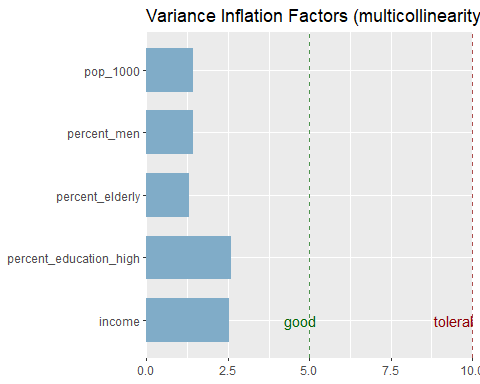


## 2.2 Model 2


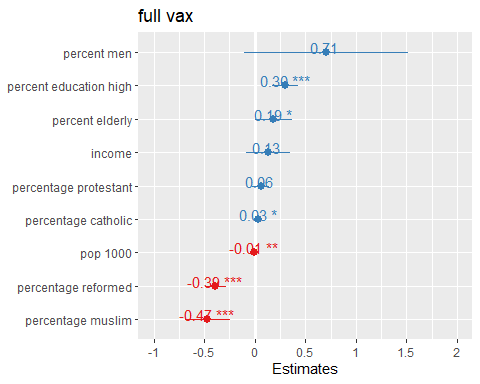


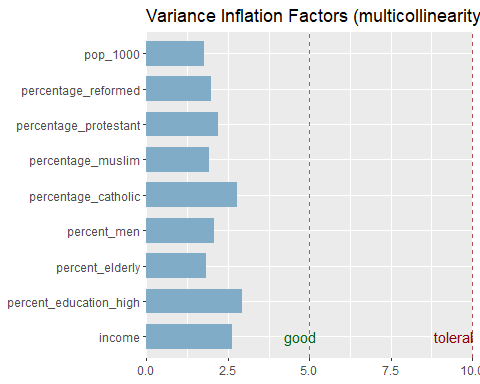


## 2.3 Model 3


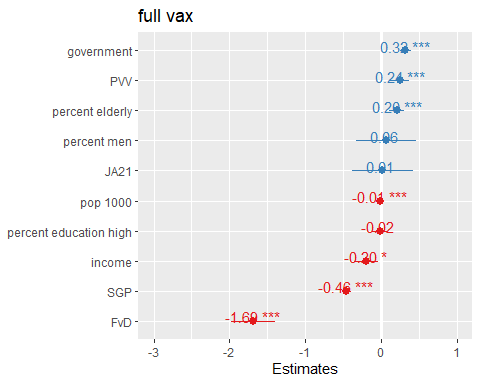


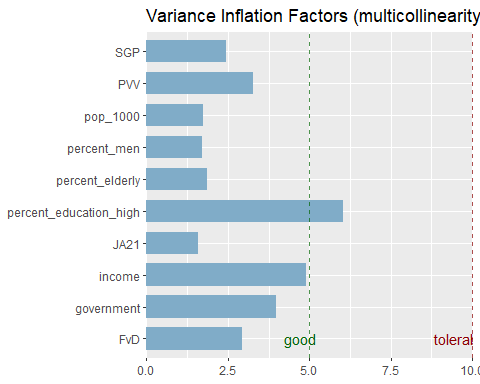


## 2.4 Model 4


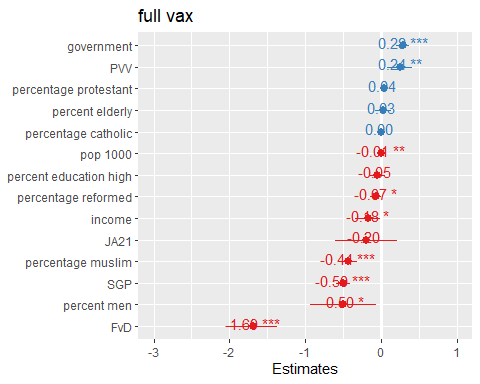


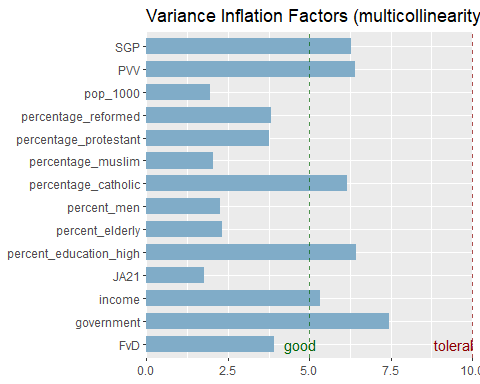


# 3 Robustness check

## 3.1 Specification curve

We can see that some of the variables in the model are highly correlated when we check for multicollinearity. Therefore, we decided to run a specification curve analysis, in which we test many possible combinations of our independent variables to make sure our results are not artifacts of the high collinearity. In total, if we estimated all potential combinations of our dependent variables we would end up with +40 million models. Since this seems like an extreme number, we randomly sampled a million models out of all possible combinations and estimated them instead. The results should not differ when compared to the full set of possible specifications of the model since we drew a random sample.

The title for each of the following graphs shows the variable for which we ran specification curve analyses. The x-axis shows the index for each specification. The y-axis shows the estimated coefficient for each model specification, blue ribbons signify positive significant effects and red negative significant coefficients (alpha < 0.05), grey ribbons show non-significant coefficients. If a specification curve is predominantly situated either below or above 0 we can be more certain of the result as it is robust to multicollinearity.

The majority of our findings are replicated using different specifications. The replication for SGP and FvD vote share are especially striking, showing that the vast majority of specifications show a negative effect on vaccination rates. However, we find that the share of men in a municipality is associated with a lower vaccination rate in a majority of specifications, whereas income, share of reformed protestants, as well as PVV and JA21 vote shares have no robust effect on vaccination rates in either direction.

## [[1]]


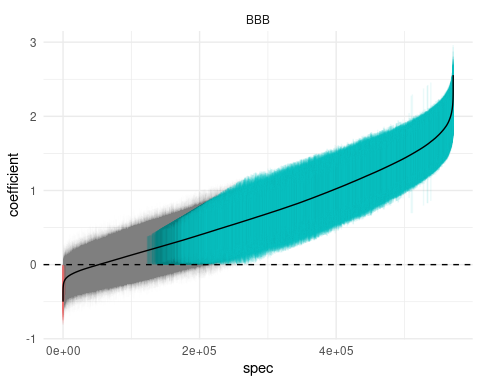


##
## [[2]]


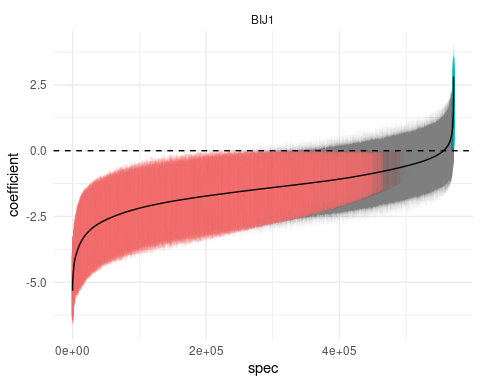


##
## [[3]]


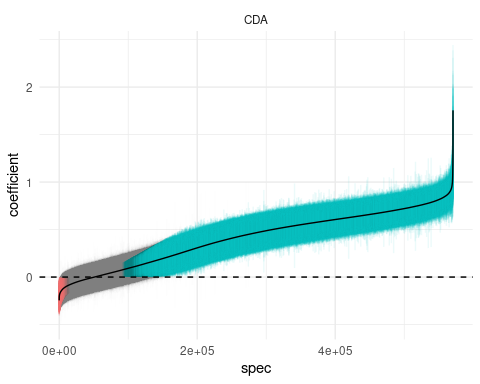


##
## [[4]]


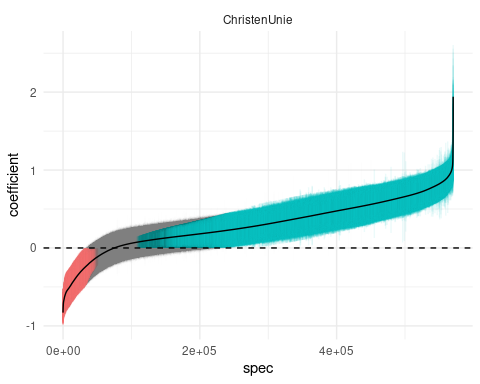


##
## [[5]]


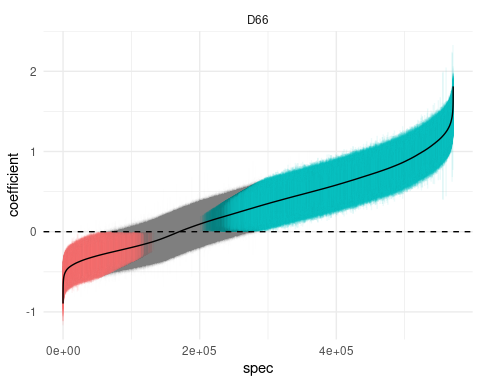


##
## [[6]]


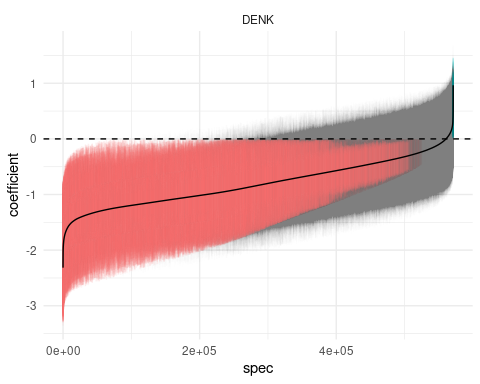


##
## [[7]]


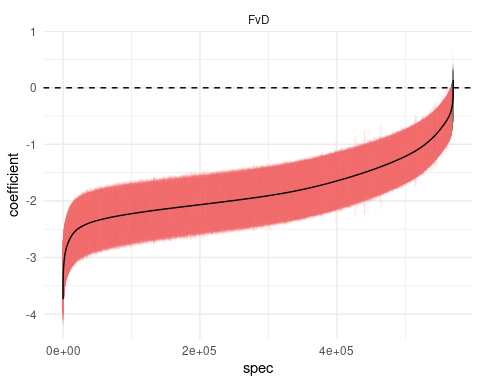


##
## [[8]]


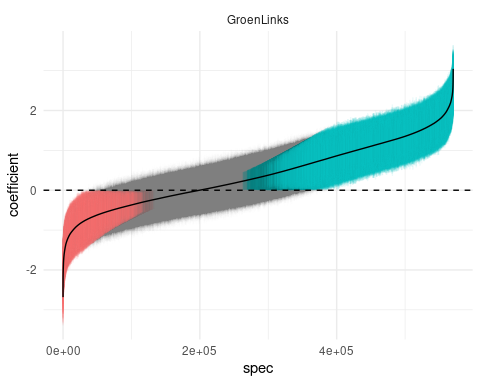


##
## [[9]]


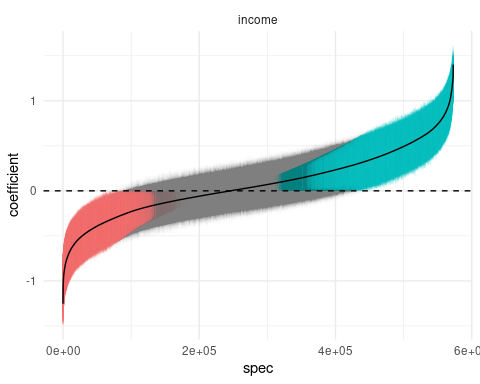


##
## [[10]]


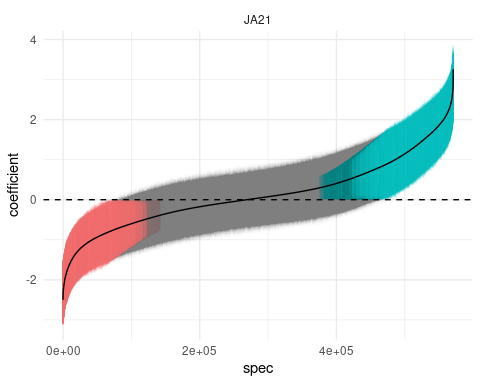


##
## [[11]]


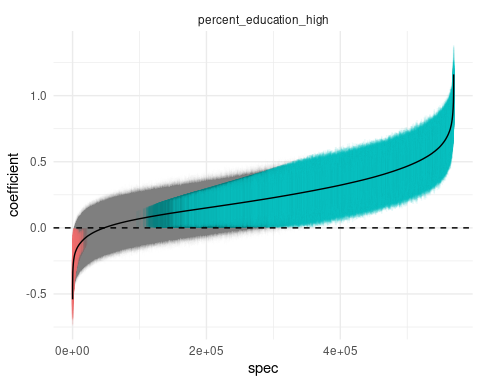


##
## [[12]]


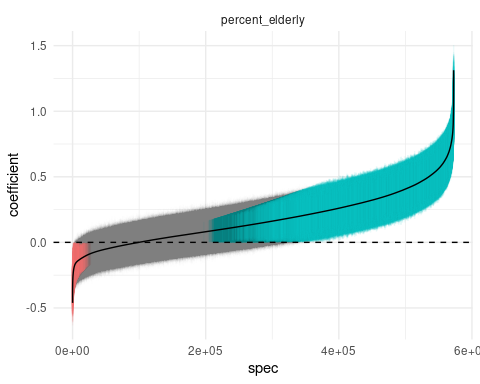


##
## [[13]]


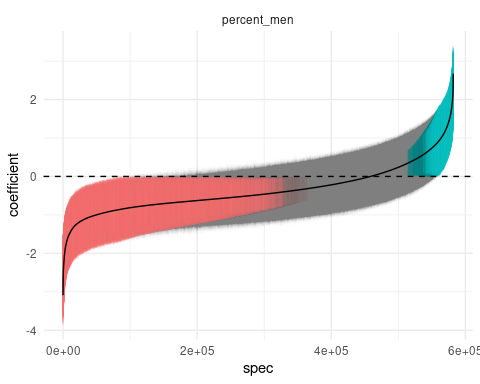


##
## [[14]]


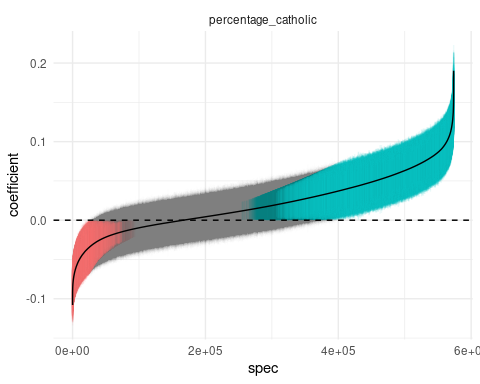


##
## [[15]]


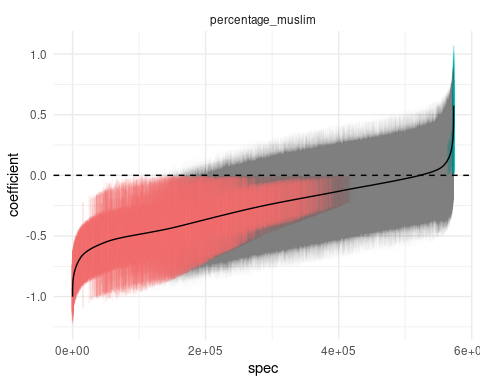


##
## [[16]]


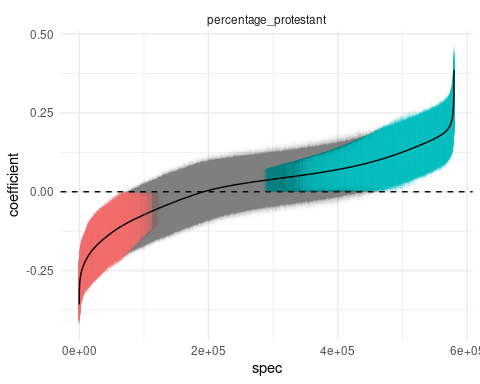


##
## [[17]]


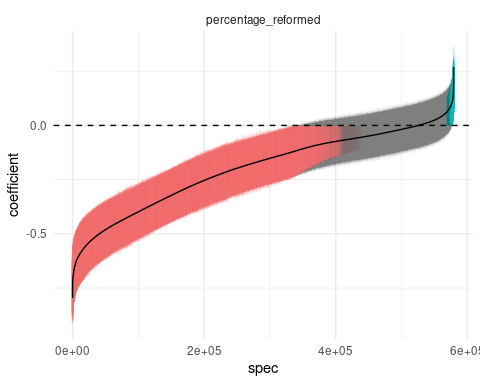


##
## [[18]]


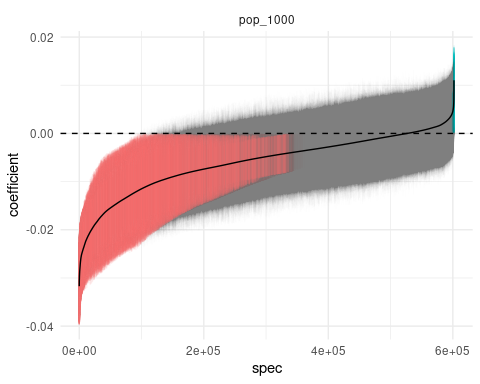


##
## [[19]]


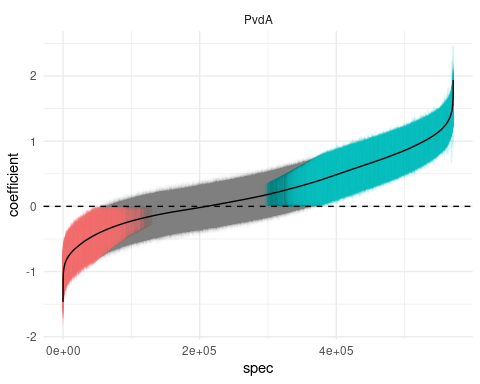


##
## [[20]]


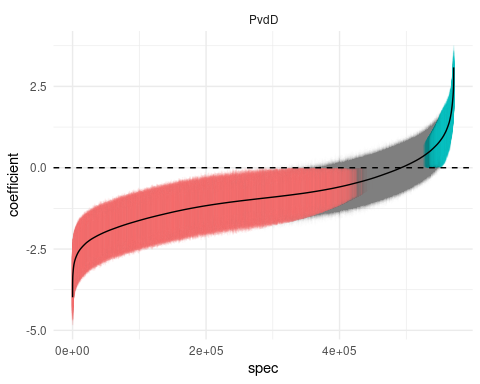


##
## [[21]]


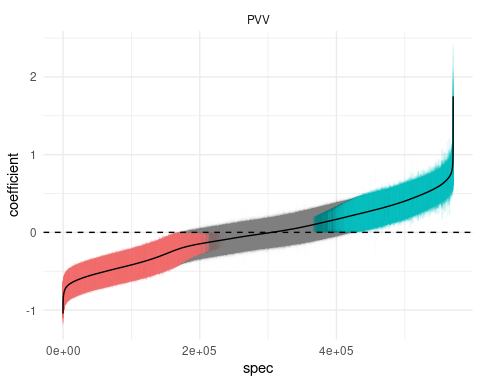


##
## [[22]]


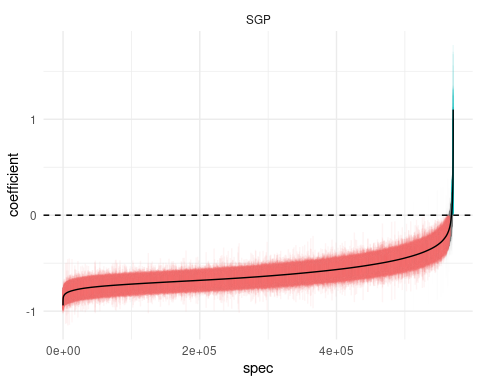


##
## [[23]]


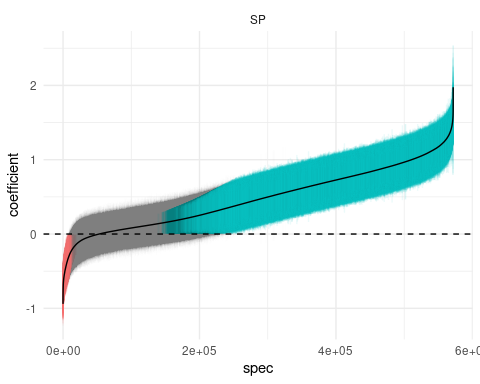


##
## [[24]]


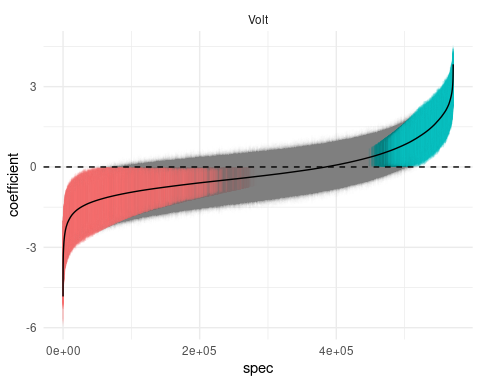


##
## [[25]]


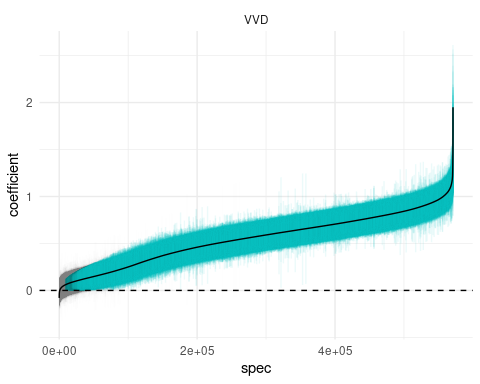


##
## [[26]]


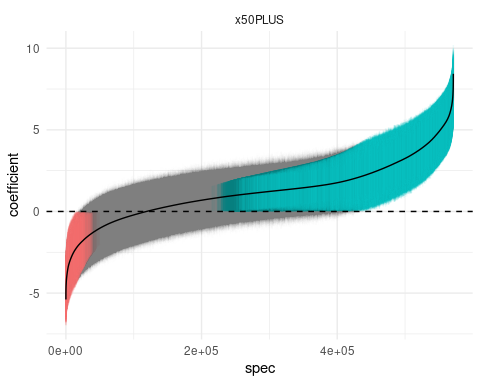

Supplement: ckac112_Supplementary_Data [file ckac112_supplementary_data.docx]
